# Supplementary material for: The Lin28b/Wnt5a axis drives pancreas cancer through crosstalk between cancer associated fibroblasts and tumor epithelium
Source: Nat Commun. 2023 Oct 28;14:6885. doi: 10.1038/s41467-023-42508-8 (PMC10613206; doi:10.1038/s41467-023-42508-8)
Supplement: Supplementary file 1 — Supplementary Information [file 41467_2023_42508_MOESM1_ESM.pdf]

Supplementary Information

The Lin28b/Wnt5a axis drives pancreas cancer through crosstalk between cancer associated fibroblasts and tumor epithelium

Zhaoqi Shu et al.

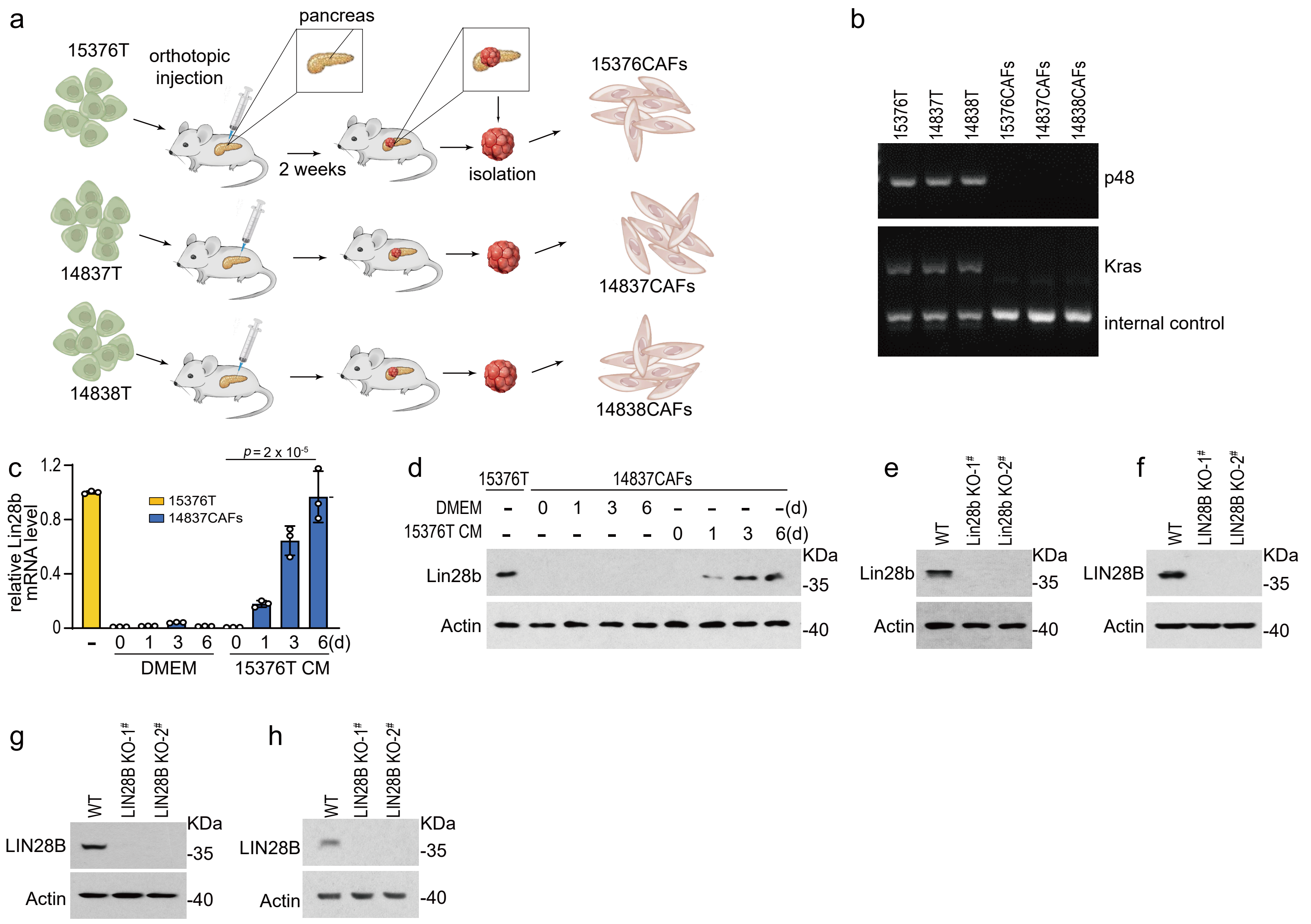

Supplementary Fig.1 Lin28b expression in CAFs is induced by conditioned medium from Lin28b^{high} tumor cells.

(a) 14837T, 14838T, or 15376T cells were orthotopically injected into C57BL/6J mice. After 2 weeks, mCAFs (14837CAFs, 14838CAFs and 15376CAFs) were isolated from the indicated tumors. (b) The genotype of 14837T, 14838T, 15376, 14837CAFs, 14838CAFs and 15376CAFs. (c-d) 14837CAFs were cultured with DMEM or 15376T-CM for 6 days. Then, Lin28b levels were measured by real-time qPCR (c) and western blotting (d). Lin28b levels in 15376T were included as a positive control. Representative of n=3 independent experiments (d). (e-h) Western blotting was used to detect the knockout efficiency of Lin28b in 15376T (e), PANC-1 (f), PANC03.27 (g) and hPDAC1# (h). Representative of n=2 independent experiments.Three biologically independent experiments were performed (c). Data are shown as mean ± s.d. P-value were determined by one-way ANOVA with Tukey's multiple comparison test (c).

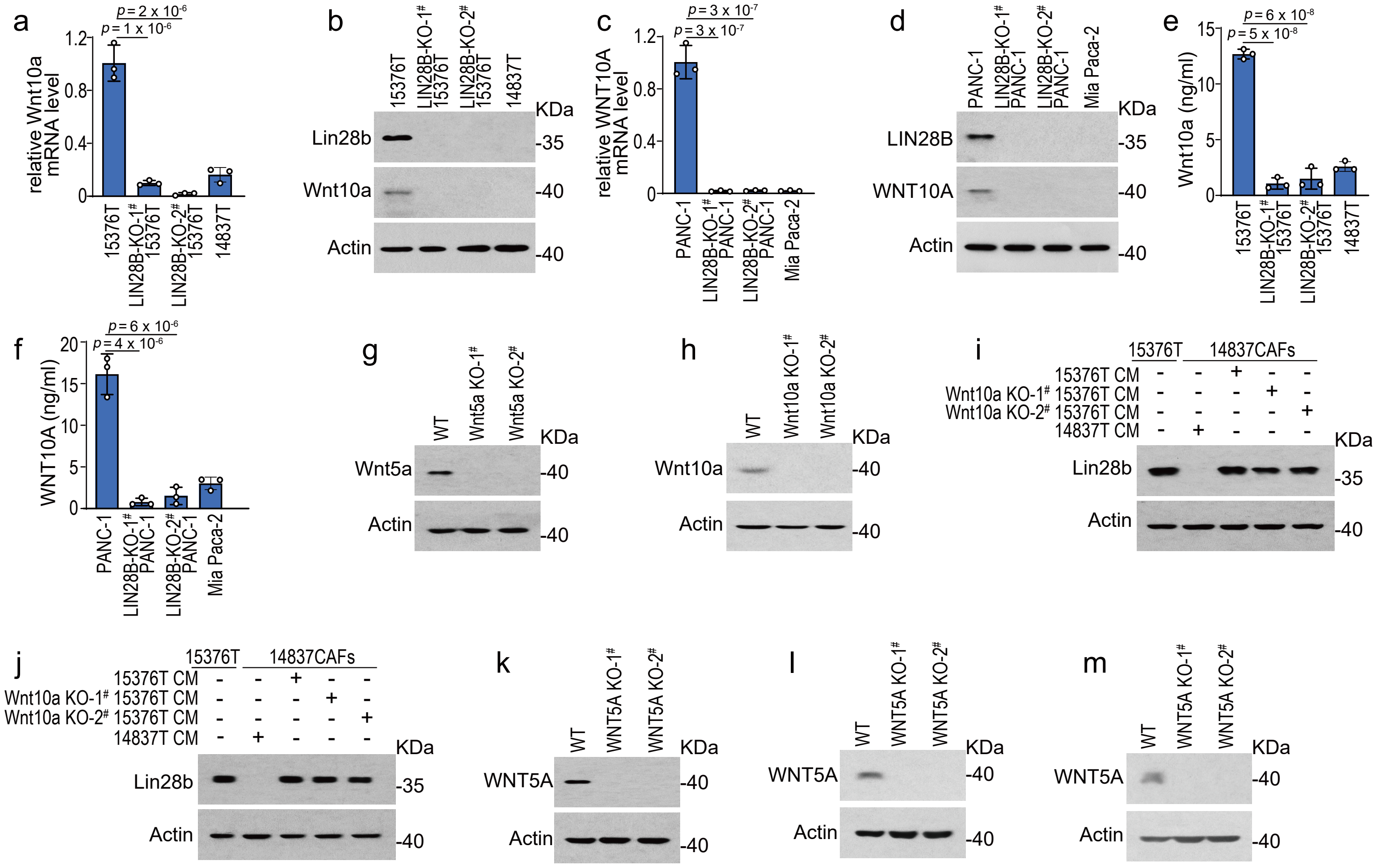

Supplementary Fig.2 Lin28b^{high} PDAC cells secret Wnt5a to induce Lin28b expression in CAFs.

(a-b) The levels of Wnt10a in 15376T, Lin28b-KO 15376T and 14837T were measured by real-time qPCR (a) and western blotting (b). Representative of n = 2 independent experiments. (c-d) The levels of WNT10A in PANC-1, LIN28B-KO PANC-1 and Mia Paca-2 were measured by real-time qPCR (c) and western blotting (d). Representative of n = 2 independent experiments. (e) Wnt10a levels in the supernatants of 15376T, Lin28b-KO 15376T and 14837T were examined by ELISA. (f) WNT10A levels in the supernatants of PANC-1, LIN28B-KO PANC-1 and Mia Paca-2 were examined by ELISA. (g-h) Western blotting was used to detect the knockout efficiency of Wnt5a (g) and Wnt10a (h) in 15376T. Representative of n = 2 independent experiments. (i-j) 14837CAFs (i) or 15376CAFs (j) were cultured with 15376T-CM or Wnt10a-KO 15376T-CM for 6 days. Then, Lin28b levels were measured by western blotting. Lin28b level in 15376T were included as a positive control. Representative of n = 3 independent experiments. (k-m) Western blotting was used to detect the knockout efficiency of WNT5A in PANC-1 (k), PANC03.27 (l) and hPDAC1# (m). Representative of n=2 independent experiments. Three biologically independent experiments were performed (a, c, e, f). Data are shown as mean±s.d. P-value were determined by one-way ANOVA with Tukey's multiple comparison test (a, c, e, f).

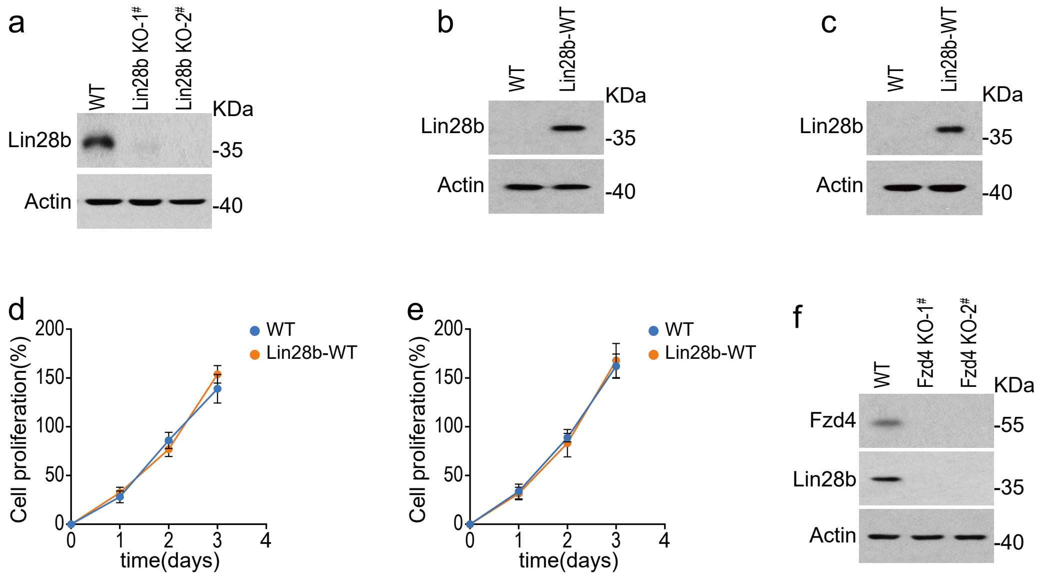

Supplementary Fig.3 Lin28b-positive CAFs promote growth of pancreatic cancer.

Before the experiment started, CAFs were cultured with 15376T-CM for 6 days to induce Lin28b expression. CM for CAFs culture was replaced daily (a, d). (a) Western blotting was used to detect the knocking-out efficiency of Lin28b in 15376CAFs. Representative of n = 2 independent experiments. (b-c) Lin28b-WT was stable expressed in 14837CAFs (b) and 15376CAFs (c). Western blotting was used to detect Lin28b expression efficiency. Representative of n=2 independent experiments. (d) 14837CAFs or Lin28b-WT 14837CAFs were cultured for 1, 2, or 3 days. The cells were counted to calculate the cell proliferation. (e) 15376CAFs or Lin28b-WT 15376CAFs were cultured for 1, 2, or 3 days. The cells were counted to calculate the cell proliferation. (f) Western blotting was used to detect the knocking-out efficiency of Fzd4 in 15376CAFs. Representative of n = 2 independent experiments.Three biologically independent experiments were performed (d, e). Data are shown as mean ± s.d. P-value were determined by one-way ANOVA with Tukey’s multiple comparison test (d, e).

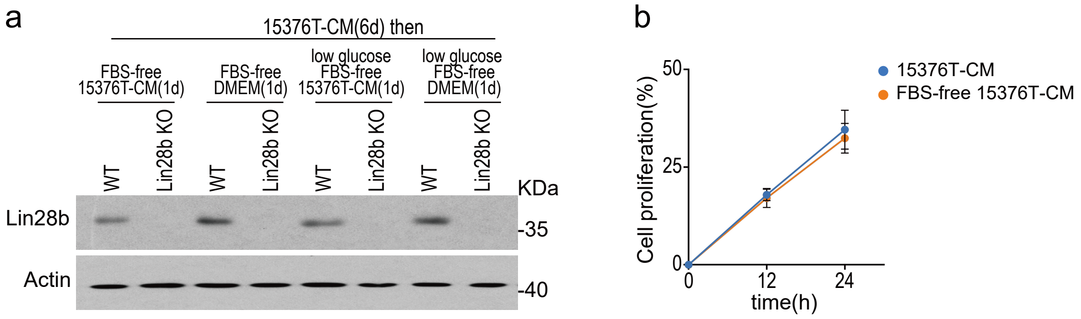

Supplementary Fig.4 Pcsk9 secreted by CAFs promotes PDAC growth.

(a) 15376CAFs and Lin28b-KO 15376CAFs were cultured with 15376T-CM for 6 days. Then, they were cultured with FBS-free 15376T-CM, FBS-free DMEM, low glucose FBS-free 15376T-CM or low glucose FBS-free DMEM. After 1 day, the levels of Lin28b were measured by western blotting. Representative of n = 2 independent experiments. (b) 15376CAFs were cultured with 15376T-CM or FBS-free 15376T-CM for 24 h. The cells were counted to calculate the cell proliferation. Three biologically independent experiments were performed (b). Data are shown as mean ± s.d. P-value were determined by one-way ANOVA with Tukey's multiple comparison test (b).

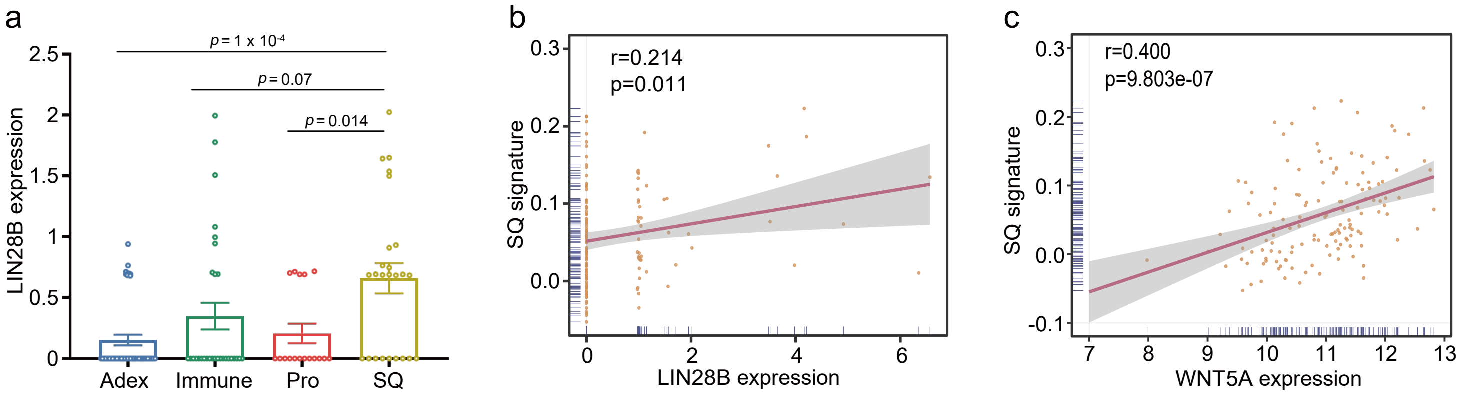

Supplementary Fig.5 Correlation between Lin28b-Wnt5a axis and squamous subtype tumors.

(a) LIN28B expression among human PDAC subtypes in PDAC RNA-seq dataset (dbGaP Study Accession: phs001287); n = 140 patients. ADEX, aberrantly differentiated endocrine exocrine subtype; Immu, immunogenic subtype; Pro, progenitor subtype; SQ, squamous subtype. P-value were determined by one-way ANOVA with Tukey's multiple comparison test. (b) Correlation between LIN28B expression and squamous subtype signature in PDAC RNA-seq dataset (dbGaP Study Accession: phs001287); n = 140 patients. (Pearson product-moment correlation test; r=0.214, p=0.011). (c) Correlation between WNT5A expression and squamous subtype signature in PDAC RNA-seq dataset (dbGaP Study Accession: phs001287); n = 140 patients. (Pearson product-moment correlation test; r=0.400, p=9.803e-07).

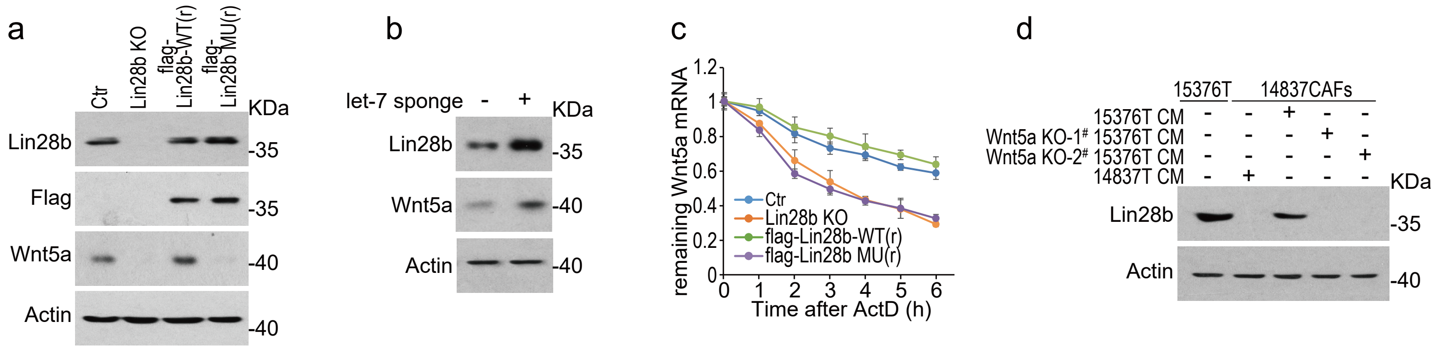

Supplementary Fig.6 Wnt5a was regulated by Lin28b/let-7 pathway.

(a) The levels of Wnt5a in 15376T, Lin28b-KO 15376T, flag-Lin28b-WT(r)-expressing 15376T and flag-Lin28b-MU(r)-expressing 15376T were measured by western blotting. Representative of n = 3 independent experiments. (b) 15376T were transfected with let-7 sponge vector. The protein levels of Lin28b and Wnt5a were measured by western blotting. Representative of n=3 independent experiments. (c) The mRNA stability of Wnt5a in 15376T, Lin28b-KO 15376T, flag-Lin28b-WT(r)-expressing 15376T and flag-Lin28b-MU(r)-expressing 15376T were measured by real-time qPCR (n = 3 independent experiments). Data are shown as mean ± s.d. P-value were determined by one-way ANOVA with Tukey's multiple comparison test. (d) 14837CAFs were cultured with 15376T-CM or Wnt5a-KO 15376T-CM under low glucose (2mM glucose) for 2 days. Then Lin28b levels were measured by western blotting. Lin28b levels in 15376T were included as a positive control. Representative of n = 3 independent experiments.

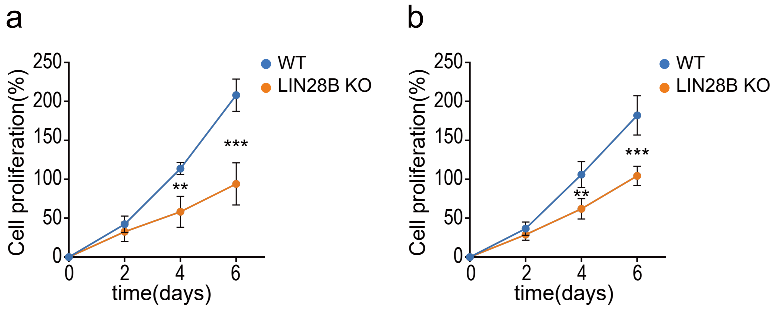

Supplementary Fig.7 Lin28b expression promoted human PDAC cell growth.

(a) PANC-1 or Lin28b-KO PANC-1 were cultured for 2, 4, or 6 days. The cells were counted to calculate the cell proliferation. (b) PANC03.27 or Lin28b-KO PANC03.27 were cultured for 2, 4, or 6 days. The cells were counted to calculate the cell proliferation. Three biologically independent experiments were performed (a, b). Data are shown as mean ± s.d. Significance levels are denoted as *p<0.05; **p<0.01; ***p<0.001.P-value were determined by one-way ANOVA with Tukey’s multiple comparison test (a, b).

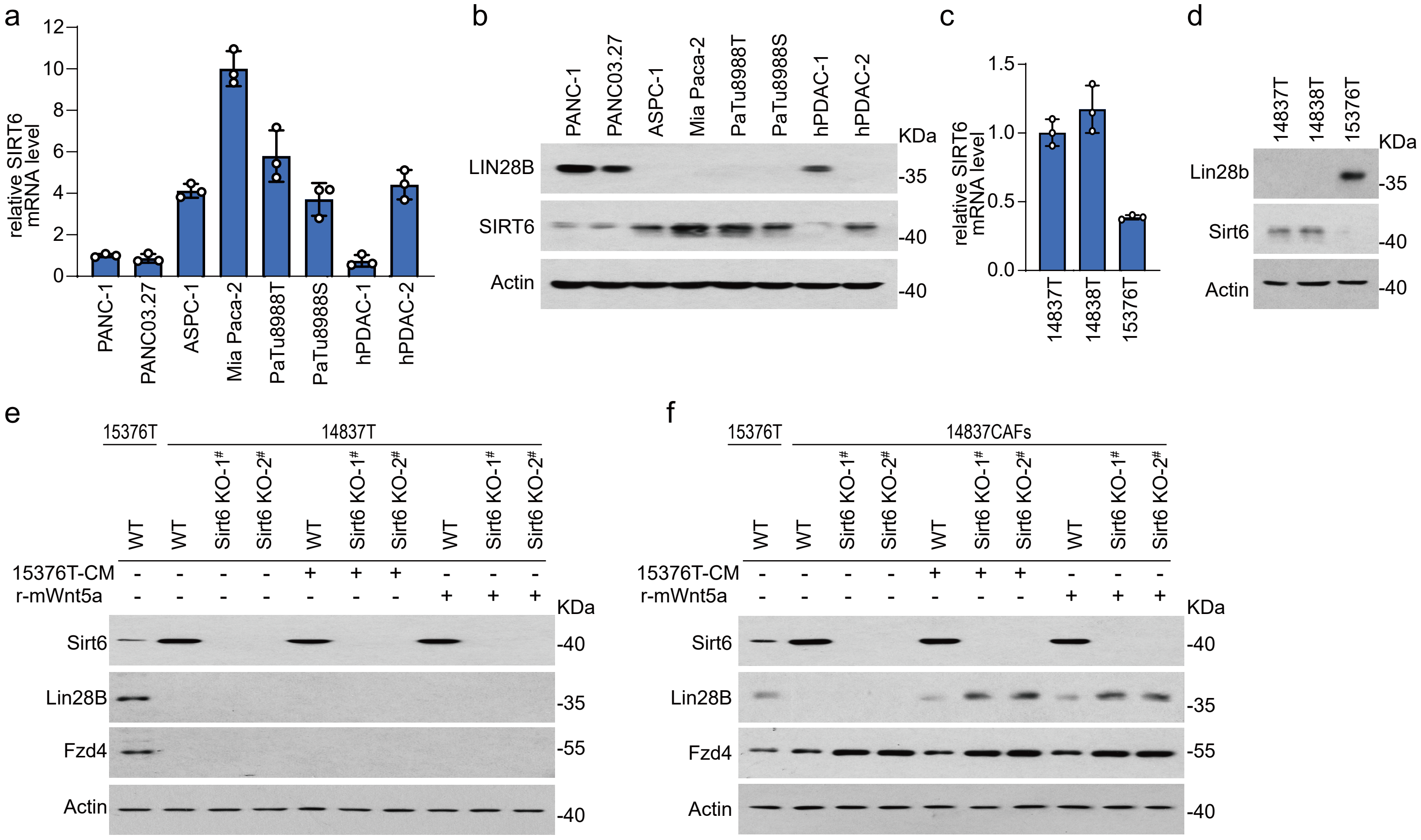

Supplementary Fig.8 The effect of Sirt6 on Lin28b expression.

(a-b) The levels of SIRT6 in human PDAC cell lines were measured by real-time qPCR (a) and western blotting (b). Representative of n = 3 independent experiments (b). (c-d) The levels of Sirt6 in mouse PDAC cell lines were measured by real-time qPCR (c) and western blotting (d). Representative of n = 3 independent experiments (d). (e-f) 14837T (e) or 14837CAFs (f) were treated with 15376T-CM or 100ng/ml r-mWnt5a for 6 days. Then, the levels of Sirt6, Lin28b and Fzd4 were measured by western blotting. Representative of n = 3 independent experiments. Three biologically independent experiments were performed (a, c). Data are shown as mean ± s.d. P-value were determined by one-way ANOVA with Tukey's multiple comparison test (a, c).

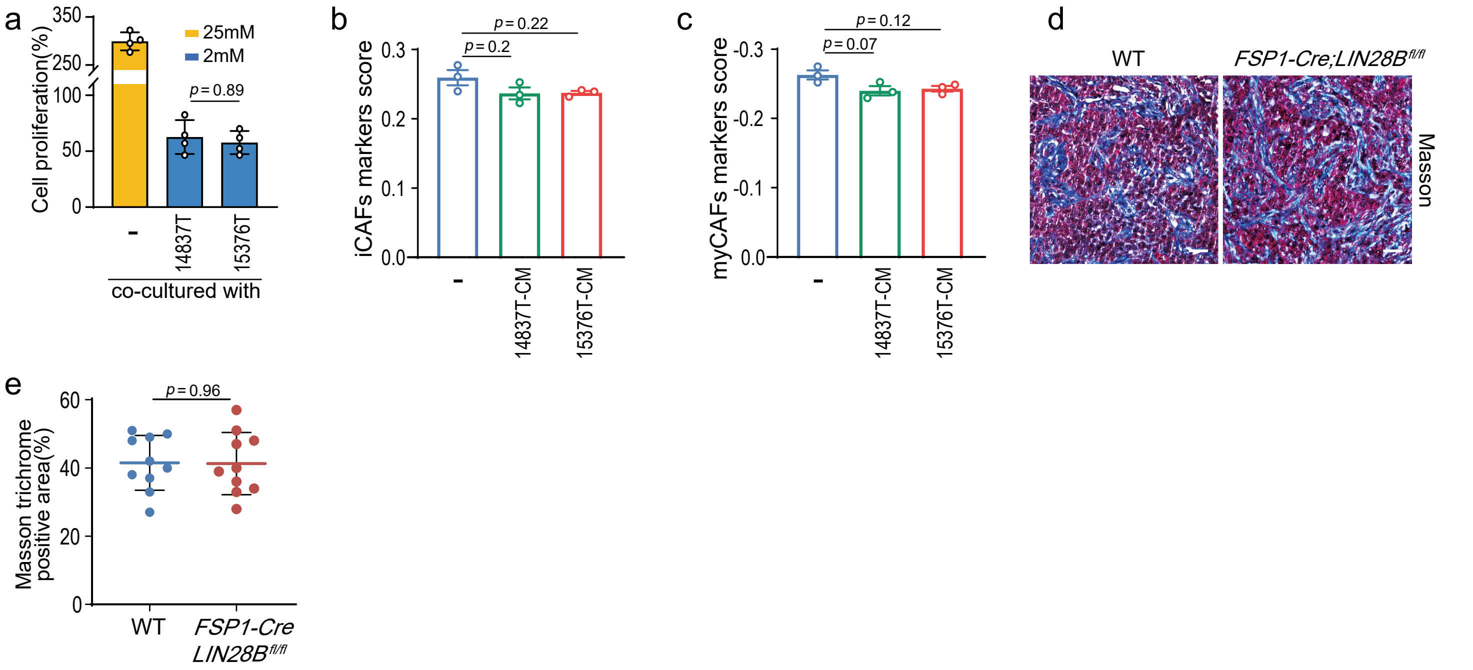

Supplementary Fig.9 Lin28b expression in tumors did not affect the survival and subtype switch of CAFs.

(a) 15376CAFs were co-cultured with 14837T or 15376T in a transwell chamber for 2 days. The cells were counted to calculate the cell proliferation. (b-c) 15376CAFs treated with 14837T-CM or 15376T-CM. iCAFs markers score (b) and myCAFs markers score (b) were shown. (d-e) 15376T cells were orthotopically injected into WT or FSP-Cre;Lin28b$^{fl/fl}$ mice (n=6 mice). After 2 weeks, the tumors were analyzed by Masson trichrome staining. Scale bars: 30 μM (d). Quantification of Masson trichrome-positive area in WT or FSP-Cre;Lin28b$^{fl/fl}$ mice (n=10 views per group) (e). Four biologically independent experiments were performed (a). Three biologically independent experiments were performed (b, c). Data are shown as mean ± s.d. P-value were determined by one-way ANOVA with Tukey's multiple comparison test (a-c) or two-tailed unpaired Student's t-tests (e).

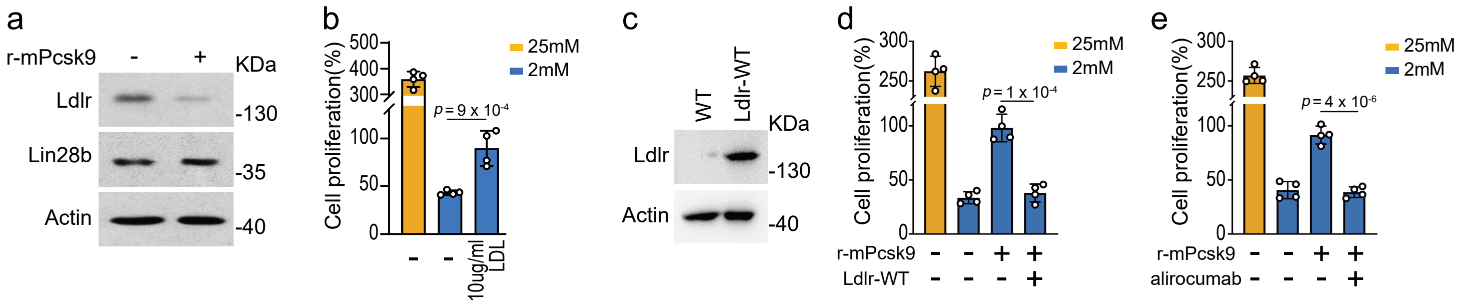

Supplementary Fig.10 Pcsk9 decreased the protein level of Ldlr.

(a) Tumors were treated with 100ng/ml r-mPcsk9 for 2 days. Then, the levels of Ldlr and Lin28b were measured by western blotting. Representative of n = 3 independent experiments. (b) 15376T were treated with 10ug/ml LDL under low glucose (2 mM) for 2 days. The cells were counted to calculate the cell proliferation. (c) Ldlr-WT was stable expressed in 15376T. Western blotting was used to detect Ldlr expression efficiency. Representative of n = 2 independent experiments. (d) 15376T and Ldlr-WT 15376T were treated with 100ng/ml recombinant Pcsk9 (r-mPcsk9) under low glucose (2 mM) for 2 days. The cells were counted to calculate the cell proliferation. (e) 15376T were treated with 100ng/ml recombinant Pcsk9 (r-mPcsk9) in the presence or absence of alirocumab (200 nM) under low glucose (2 mM) for 2 days. The cells were counted to calculate the cell proliferation. Four biologically independent experiments were performed (b, d, e). Data are shown as mean±s.d. P-value were determined by one-way ANOVA with Tukey's multiple comparison test (b, d, e).
