# Supplementary material for: The Lin28b/Wnt5a axis drives pancreas cancer through crosstalk between cancer associated fibroblasts and tumor epithelium
Source: Nat Commun. 2023 Oct 28;14:6885. doi: 10.1038/s41467-023-42508-8 (PMC10613206; doi:10.1038/s41467-023-42508-8)
Supplement: Supplementary file 2 — Description of Additional Supplementary Files [file 41467_2023_42508_MOESM2_ESM.pdf]

Description of Additional Supplementary Files

Supplementary Data 1
PDAC-Subtype Signature Genes Analysis.

Supplementary Data 2
Gene expression of iCAFs/myCAFs markers in 15376CAFs which were cultured with 14837T-CM or 15375T-CM for 6 days.

Supplementary Data 3
Patient information of Tissue microarray.

Supplementary Data 4
Quantitative secretomics analysis of 15376CAFs-CM and Lin28b-KO 15376CAFs-CM.
