# Supplementary material for: The Lin28b/Wnt5a axis drives pancreas cancer through crosstalk between cancer associated fibroblasts and tumor epithelium
Source: Nat Commun. 2023 Oct 28;14:6885. doi: 10.1038/s41467-023-42508-8 (PMC10613206; doi:10.1038/s41467-023-42508-8)
Supplement: Supplementary file 4 — Reporting Summary [file 41467_2023_42508_MOESM4_ESM.pdf]

nature portfolio

Corresponding author(s): Ying Zhao

Last updated by author(s): Sep 14, 2023

Reporting Summary

Nature Portfolio wishes to improve the reproducibility of the work that we publish. This form provides structure for consistency and transparency in reporting. For further information on Nature Portfolio policies, see our Editorial Policies and the Editorial Policy Checklist.

Statistics

For all statistical analyses, confirm that the following items are present in the figure legend, table legend, main text, or Methods section.

n/a	Confirmed	
☐	☒	The exact sample size (*n*) for each experimental group/condition, given as a discrete number and unit of measurement
☐	☒	A statement on whether measurements were taken from distinct samples or whether the same sample was measured repeatedly
☐	☒	The statistical test(s) used AND whether they are one- or two-sided<br>*Only common tests should be described solely by name; describe more complex techniques in the Methods section.*
☐	☒	A description of all covariates tested
☐	☒	A description of any assumptions or corrections, such as tests of normality and adjustment for multiple comparisons
☐	☒	A full description of the statistical parameters including central tendency (e.g. means) or other basic estimates (e.g. regression coefficient) AND variation (e.g. standard deviation) or associated estimates of uncertainty (e.g. confidence intervals)
☐	☒	For null hypothesis testing, the test statistic (e.g. *F*, *t*, *r*) with confidence intervals, effect sizes, degrees of freedom and *P* value noted<br>*Give P values as exact values whenever suitable.*
☐	☒	For Bayesian analysis, information on the choice of priors and Markov chain Monte Carlo settings
☐	☒	For hierarchical and complex designs, identification of the appropriate level for tests and full reporting of outcomes
☐	☒	Estimates of effect sizes (e.g. Cohen's *d*, Pearson's *r*), indicating how they were calculated

Our web collection on statistics for biologists contains articles on many of the points above.

Software and code

Policy information about availability of computer code

Data collection	Image collection was performed with confocal microscope (ZEISS LSM880 laser scanning confocal microscope and ZEISSZEN software).<br>Quantitative RT-PCR (qRT-PCR) results were recorded with Applied Biosystems 7500 according to the manufacturer's instructions.
Data analysis	The tissue sections stained immunohistochemically were analyzed and the mean staining intensity was calculated using Image-Pro Plus software.<br>The oligonucleotide primers for qRT-PCR were designed using Light Cycler Probe Design Software 2.0 (Roche) or Primer Bank (http://pga.mgh.harvard.edu/primerbank/index.html).<br>Statistical analysis was performed by using the SPSS statistical software package (standard version 20; SPSS Inc., Chicago, IL, USA).

For manuscripts utilizing custom algorithms or software that are central to the research but not yet described in published literature, software must be made available to editors and reviewers. We strongly encourage code deposition in a community repository (e.g. GitHub). See the Nature Portfolio guidelines for submitting code & software for further information.

Data

Policy information about availability of data

All manuscripts must include a data availability statement. This statement should provide the following information, where applicable:

- Accession codes, unique identifiers, or web links for publicly available datasets
- A description of any restrictions on data availability
- For clinical datasets or third party data, please ensure that the statement adheres to our policy

The detailed results from the RNA-Seq and WES experiments are deposited in the NCBI's Gene Expression Omnibus (GEO GSE217318).
The detailed results from the RNA-Seq and WES experiments are deposited in the ProteomeXchange Consortium via the iProX with the dataset identifier PXD045477
The detailed results from the human PDAC RNA-seq dataset from Clinical Proteomic Tumor Analysis Consortium (CPTAC) genomic data (dbGaP Study Accession: phs001287).

Research involving human participants, their data, or biological material

Policy information about studies with human participants or human data. See also policy information about sex, gender (identity/presentation), and sexual orientation and race, ethnicity and racism.

Reporting on sex and gender	The research findings apply to both male and female. Clinical sopecimens used for IHC analvsis were obtained from male patients as indicated in Supplementary Data file 3.
Reporting on race, ethnicity, or other socially relevant groupings	The PDAC samples used in this study were obtained exclusively from Chinese individuals, including both Asian male and Asian female. The tissue samples were collected from the department of pancreatic surgery, the Huashan hospital with FuDan University University, Shanghai, China. The race/ethnicity variable was not used as a covariate in this study.
Population characteristics	The hPDAC, hCAFs, human TMA samples were collected from PDAC patients(male/female) from 30-85 years old diagnosed with PDAC at department of pancreatic surgery, Huashan Hospital.
Recruitment	PDAC patients who received pancreaticoduodenectomy/total pancreatectomy/distal pancreatectomy surgery were randomly recruited into the study.Eligibility criteria for selection of the patients:<br>1. Encountered between June 2019 and December 2020<br>2. Age is not limited<br>3. Underwent surgical therapy and histologically diagnosed postoperatively by two experienced pathologists<br>4. In accordance with TNM staging system in PDAC of 8th edition AICC/UICC Classification of Malignant Tumors<br>5. Tumor tissues of stage IV were confined to those patients who underwent palliative surgery with isolated or single segmental liver metastasis.
Ethics oversight	All manipulations about human patient samples were approved by the Research Ethics Committee of Huashan Hospital, Fudan University, and all the patients were provided with written informed consent before enrolment. (protocol number:KY2020-015)

Note that full information on the approval of the study protocol must also be provided in the manuscript.

Field-specific reporting

Please select the one below that is the best fit for your research. If you are not sure, read the appropriate sections before making your selection.

- [x] Life sciences
- [ ] Behavioural & social sciences
- [ ] Ecological, evolutionary & environmental sciences

For a reference copy of the document with all sections, see nature.com/documents/nr-reporting-summary-flat.pdf

Life sciences study design

All studies must disclose on these points even when the disclosure is negative.

Sample size	No statistical method was used to predetermine sample size in all the highly controlled in vitro or in vivo experiments, but our sample sizes are similar to those reported in previous publications. For the mouse work, we aimed to have a minimum of 6 mice in each group. For the human TMA analysis, the sample size consisted of 80 TMA cores/patients. For the cell proliferation assay, the sample size consisted of 4 biologically independent experiments. For the real-time qPCR, western blotting, Chip, luciferase assay, and elisa, the sample size consisted of 3 biologically independent experiments. The exact sample size was indicated in the figure legend.
Data exclusions	No data was excluded from the analyses
Replication	The experimental findings were successfully replicated a minimum of three times with similar results
Randomization	For the in vivo experiments, littermate animals from different cages were randomly assigned to the experimental groups. For in vitro experiments, randomization was not relevant as the samples were treated and analyzed in the same manner.

Blinding	Tumor weihgt measurements were performed in a blinded manner. All other data collection and analysis were not performed blind to the conditions of the experiments,, but data were analyzed by multiple investigators, including those not involved in the experiment.

Reporting for specific materials, systems and methods

We require information from authors about some types of materials, experimental systems and methods used in many studies. Here, indicate whether each material, system or method listed is relevant to your study. If you are not sure if a list item applies to your research, read the appropriate section before selecting a response.

Materials & experimental systems

n/a	Involved in the study
☐	☒ Antibodies
☐	☒ Eukaryotic cell lines
☒	☐ Palaeontology and archaeology
☐	☒ Animals and other organisms
☒	☐ Clinical data
☒	☐ Dual use research of concern
☒	☐ Plants

Methods

n/a	Involved in the study
☒	☐ ChIP-seq
☒	☐ Flow cytometry
☒	☐ MRI-based neuroimaging

Antibodies

Antibodies used	The antibodies used were anti-Lin28B (Abcam, #ab191881,1:1000; abcepta, #AP1485C, 1:1000), anti-actin (Santa Cruz, #sc-7210, 1:1000), anti-Wnt5a (Abcam, #ab229200, 1:500; R&D, #MAB645, 1:200), anti-Wnt10a (ABclonal, #A15602, 1:1000; biodragon, #BD-PNO283, 1:1000), anti-β-catenin (Abcam, #ab32572, 1:10000), anti-Pcsk9 (Abcam, #ab185194, 1:1000), anti-PGRN (Abcam, #ab187070, 1:1000), and anti-Ldlr (Abcam, #ab52818, 1:500) for WB; anti-Ki67 (Abcam, #ab16667, 0.03ug/ml), anti-α-SMA (Gene Tech, #GM085129, 1:100), anti-CK19 (Proteintech, #10712-1-AP, 0.5ug/ml), anti-Lin28B (Abcam, #ab191881,1:50; abcepta, #AP1485C, 1:50) and anti-Pcsk9 (Abcam, #ab185194, 1:100) for IF and IHC; anti-Fzd4 (R&D Systems, #MAB194, 1:100), rabbit anti-rat IgG-PE (Solarbio, #K0032R-PE, 1:100), anti-CD45-AlexaFluor 647 (BioLegend, #103124, 1:200), anti-CD326 (Ep-CAM)-AlexaFluor 488 (BioLegend, #118212, 1:25), anti-CD31-AlexaFluor 647 (BioLegend, #102416, 1:200) and anti-PDPN-APC/Cy7 (BioLegend, #127418, 1:200) for FACS.
Validation	The antibodies were validated by WB with human/mice proteins from KO or KD cell lines.

Eukaryotic cell lines

Policy information about cell lines and Sex and Gender in Research

Cell line source(s)	Primary mouse PDAC lines 14837T, 14838T and 15376T were isolated from genetically engineered C57BL/6 mice (tetO_LKrasG12D, p53 L/+, p48-Cre). 8988T, 8988S, MIA PaCa-2, ASPC1, PANCO3.27 and PANC-1 cells are human pancreatic cancer cell lines (from ATCC). 293T was purchased from ATCC. mCAFs were generated from C57BL/6J harbouring mouse PDAC. hCAFs were generated from PDAC patients.
Authentication	Primary mouse PDAC lines 14837T and 15376T were verified by genotyping and WES. CAFs were verified by measuring aSMA expression. All commercially available cell lines were analyzed by STR analysis.
Mycoplasma contamination	All cell lines are negative for mycoplasma contamination
Commonly misidentified lines (See ICLAC register)	No commonly misidentified cell lines were used in the study

Animals and other research organisms

Policy information about studies involving animals; ARRIVE guidelines recommended for reporting animal research, and Sex and Gender in Research

Laboratory animals	Six-week-old C57BL/6J mice (male/female) were used for tumorigenesis. Mice for fibroblast deletion of LIN28B were generated by breeding LIN28Bfl/fl mice with FSP-Cre mice (Model Organisms Centre, Shanghai). LIN28Bfl/fl mice were ordered from Cyagen Biosciences (Suzhou, China).Six-week-old LIN28Bfl/fl;FSP-Cre mice (male/female) were used for tumorigenesis. Mice (male/female) received standard chow diet ad libitum and were housed in conditions of 12/12h dark/light cycle, 22±1C ambient temperature and 50±10% humidity at Peking University Health Science Center animal facility. Sex was not considered in our study design.Maximal tumor burden (10% of body weight) and maximal tumor size (2.0cm) allowed by the ethics committee was not exceeded.
Wild animals	the study did not involve wild animals
Reporting on sex	The research findings apply to both male and female.

Field-collected samples	the study did not involve samples from the field
Ethics oversight	All animal experiments were performed in accordance with a protocol approved by the Department of Laboratory Animal Science oflPeking University Health Science Center and supervised by the institutional review board of Peking University.

Note that full information on the approval of the study protocol must also be provided in the manuscript.
